# Tunable longitudinal modes in extended silver nanoparticle assemblies

**DOI:** 10.3762/bjnano.7.113

**Published:** 2016-08-26

**Authors:** Serene S Bayram, Klas Lindfors, Amy Szuchmacher Blum

**Affiliations:** 1 Department of Chemistry, McGill University, 801 Sherbrooke Street West, Montreal, QC H3A 0B8, Canada; 2 Department of Chemistry, University of Cologne, Luxemburger Str. 116, 50939 Köln, Germany

**Keywords:** plasmon coupling, self-assembly, silver nanoparticles

## Abstract

Nanostructured materials with tunable properties are of great interest for a wide range of applications. The self-assembly of simple nanoparticle building blocks could provide an inexpensive means to achieve this goal. Here, we generate extended anisotropic silver nanoparticle assemblies in solution using controlled amounts of one of three inexpensive, widely available, and environmentally benign short ditopic ligands: cysteamine, dithiothreitol and cysteine in aqueous solution. The self-assembly of our extended structures is enforced by hydrogen bonding. Varying the ligand concentration modulates the extent and density of these unprecedented anisotropic structures. Our results show a correlation between the chain nature of the assembly and the generation of spectral anisotropy. Deuterating the ligand further enhances the anisotropic signal by triggering more compact aggregates and reveals the importance of solvent interactions in assembly size and morphology. Spectral and morphological evolutions of the AgNPs assemblies are followed via UV–visible spectroscopy and transmission electron microscopy (TEM). Spectroscopic measurements are compared to calculations of the absorption spectra of randomly assembled silver chains and aggregates based on the discrete dipole approximation. The models support the experimental findings and reveal the importance of aggregate size and shape as well as particle polarizability in the plasmon coupling between nanoparticles.

## Introduction

In recent years, there has been significant progress in the synthesis of nanoparticles of controlled composition, shape, and size. However, interesting nanoparticle properties often depend on the proximity relationships between neighbouring particles in addition to the composition of the building blocks themselves [1–2]. A wide range of self-assembly strategies have been used to assemble nanoparticles into organized nanostructures [1–4]. Template-based or directed assemblies result in structures dictated by template size and shape. Both soft templates such as polymers [5], DNA [6], and proteins [7], including virus coat proteins [8–9] as well as rigid templates such as carbon nanotubes [10] have been extensively implemented and programmed for desired assemblies. In addition, assisted organization and alignment of nanoparticles via external directing magnetic [11] and electric [12] fields have also been reported. Such methods depend on the inherent pinned magnetic moments and induced polarizability of the particles, respectively. Finally, interfacial techniques such as Langmuir–Blodgett films [13–14] and evaporation-induced self-assembly [15] have also been used to assemble nanostructures at interfaces.

The approach described here, in-solution self-assembly of nanostructures, can generate chains [16], sheets [17], 3D crystals [18–19] and more complex architectures [20]. In this approach, assembly is governed by a balance between attractive and repulsive interactions between the nanoparticles themselves. This approach employs a rational choice of solvent [17], a specific design of linker molecules [18] and/or a selective modification of crystal faces of nanoparticles [16] in order to assemble the target structure. Despite the success of the aforementioned methods in initiating small assemblies, larger-scale assemblies have not yet been achieved, perhaps due to the relatively large linker molecules utilized, which might restrict the nanoparticles mobility due to size and solubility matters. Interestingly though, such assemblies can be reversible, and can be triggered or directed by solution conditions such as temperature, illumination, pH and metal ion concentration [21].

Amongst the various self-assembled nanostructures, silver nanoparticles (AgNPs) are of great significance due to their sharp plasmon resonance, the antimicrobial function and a distinguished Raman spectroscopic enhancement [22]. Although AgNPs have been assembled into higher-order structures via multiple strategies [23–26], none of the resulting structures exhibit highly anisotropic spectral signatures, i.e., well-resolved isotropic/axial and anisotropic/longitudinal SPR bands that mimic their equivalents in anisotropic isolated uncoupled particles such as nanoprisms [27] and nanorods [28–29]. In addition, attempts to chain the particles can induce unwanted soldering [23] upon UV irradiation or uneven heating when microwave irradiation is used [26]. The latter method also fails for larger-scale synthesis due to technical limitations. Other attempts such as assembling on templates of λ-DNA networks have not demonstrated the ability to generate discrete plasmonic modes, and, since they are substrate-based, lack the versatility of tuning the plasmonic bands [24]. Surfactant-mediated assembly of AgNPs requires prolonged incubation times without generating intense anisotropy [25]. Herein, we report a “green & cheap” kit for the facile and quick assembly of AgNPs into highly anisotropic structures that reveal chain-like, branched networks as well as dense aggregates mediated by hydrogen-bonding short ligands that are known to well passivate the AgNPs surface [30]. We report the substantial influence of the ligand-to-particle ratio on the aggregate configuration and its optical signal. We observe a large influence of NP ensemble size and inter-nanoparticle spacing on the emergence of a well-defined bimodal spectral response. These parameters can be chemically manipulated, resulting in tunable optical properties throughout the visible range.

The optical properties of metal nanoparticles are primarily derived from interactions between electromagnetic waves and delocalized valence electrons. For example, localized plasmon resonance arises from the restoring force exerted on electrons driven by an external field, which results in field amplification in the near-field zone at the particle surface. Alterations in particle size and shape cause a frequency shift in the localized surface plasmon resonance away from the Fröhlich frequency, which defines the dipole surface plasmon of an isolated nanoparticle. Additional frequency shifts arise in particle ensembles due to electromagnetic interactions and coupling between the localized modes. Subsequent highly confined fields, also termed “hot spots”, in the junctions of closely spaced particles in ordered and disordered arrays also enable fluorescent emission enhancement [31–32]. Gold (AuNPs) and AgNPs exhibit plasmon resonance in the visible window of the electromagnetic spectrum, rendering them excellent candidates for a number of modern applications in surface-enhanced Raman spectroscopy (SERS), optical sensing and emission enhancement of molecules residing in the near field [33–34].

In addition to applications in spectroscopy, plasmonic interactions may also be exploited in other light-based devices. The miniaturization of photonic structures is essential to increase the sensitivity and speed of photonic devices [35–36]. Common dielectric waveguides are restricted by the diffraction limit of light, which, for visible light, is several hundreds of nanometres. For nanodevices, much smaller photonic elements are sought through extended planar structures capable of light guiding. The flux of surface plasmons can be tuned and acclimatized for a desired purpose by the controlled organization of metallic nanoparticles into higher order arrays and assemblies.

## Results and Discussion

**Figure 1:**
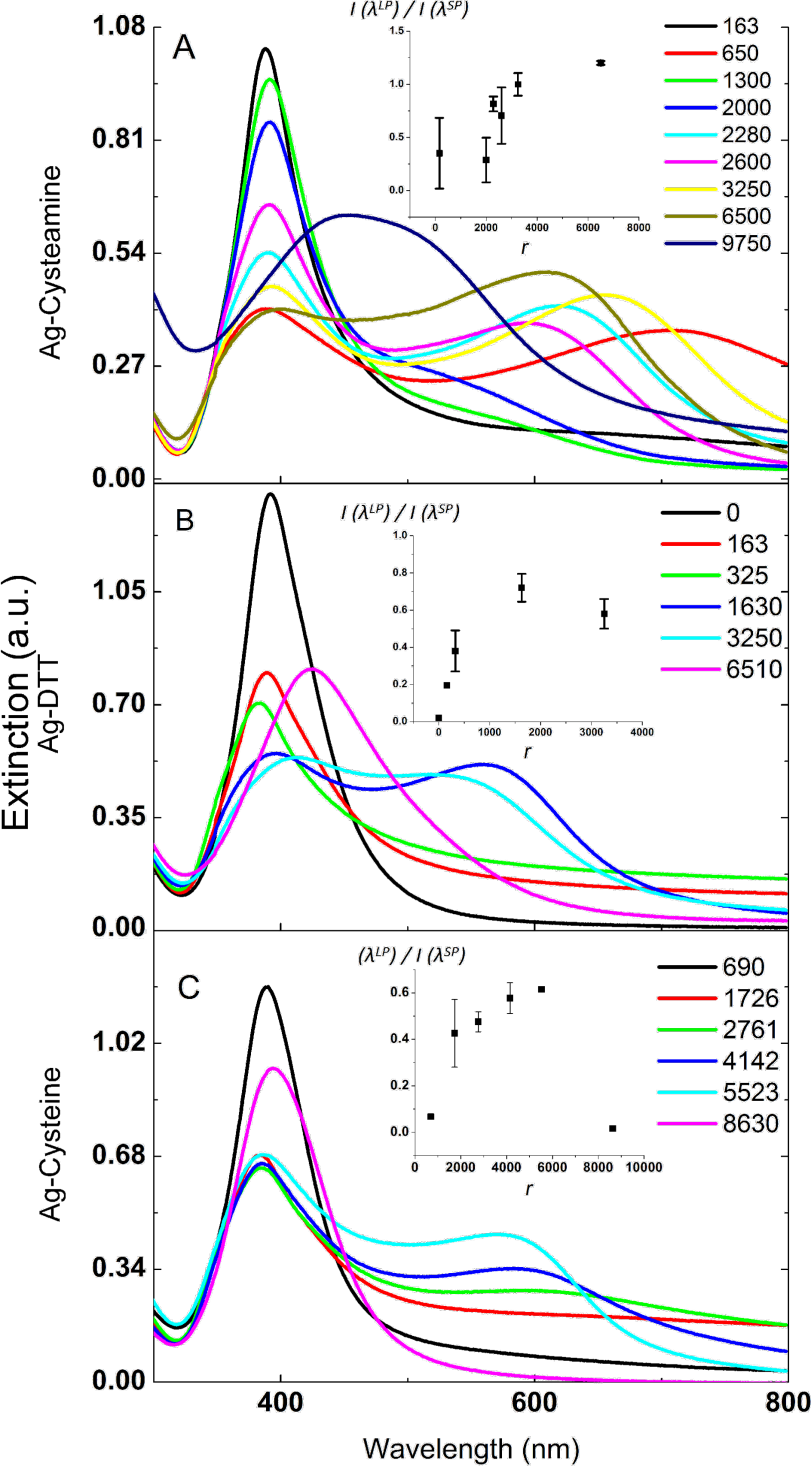
Extinction spectra of AgNPs modified by varying ratios of ligands: A) cysteamine, B) DTT and C) cysteine. Legend: *r* = number of ligand molecules/AgNP. Insets show the growth of the longitudinal plasmon band (LP) relative to the transverse band (SP) as a function of *r*. Error bars represent the standard deviations of independent spectral measurements from the average value for every ligand ratio. The most intense anisotropy is noticed in the case of cysteamine-modified AgNPs with a ratio exceeding unity at 5000 < *r* < 9000.

The as-synthesized AgNPs do not show any sign of assembly, and have an average size of 13.9 nm (Supporting Information File 1, Figure S1A). The addition of the experimental ligands triggers assembly, as shown by changes in the extinction spectra that depend on the ligand to nanoparticle ratio. Figure 1 shows the extinction spectra of AgNPs modified by increasing amounts of the three H-bonding ligands: cysteamine, dithiothreitol (DTT) and cysteine. All spectra show a band at 398–410 nm, corresponding to the expected local plasmon resonance for spherical silver nanoparticles of this size.

**Figure 2:**
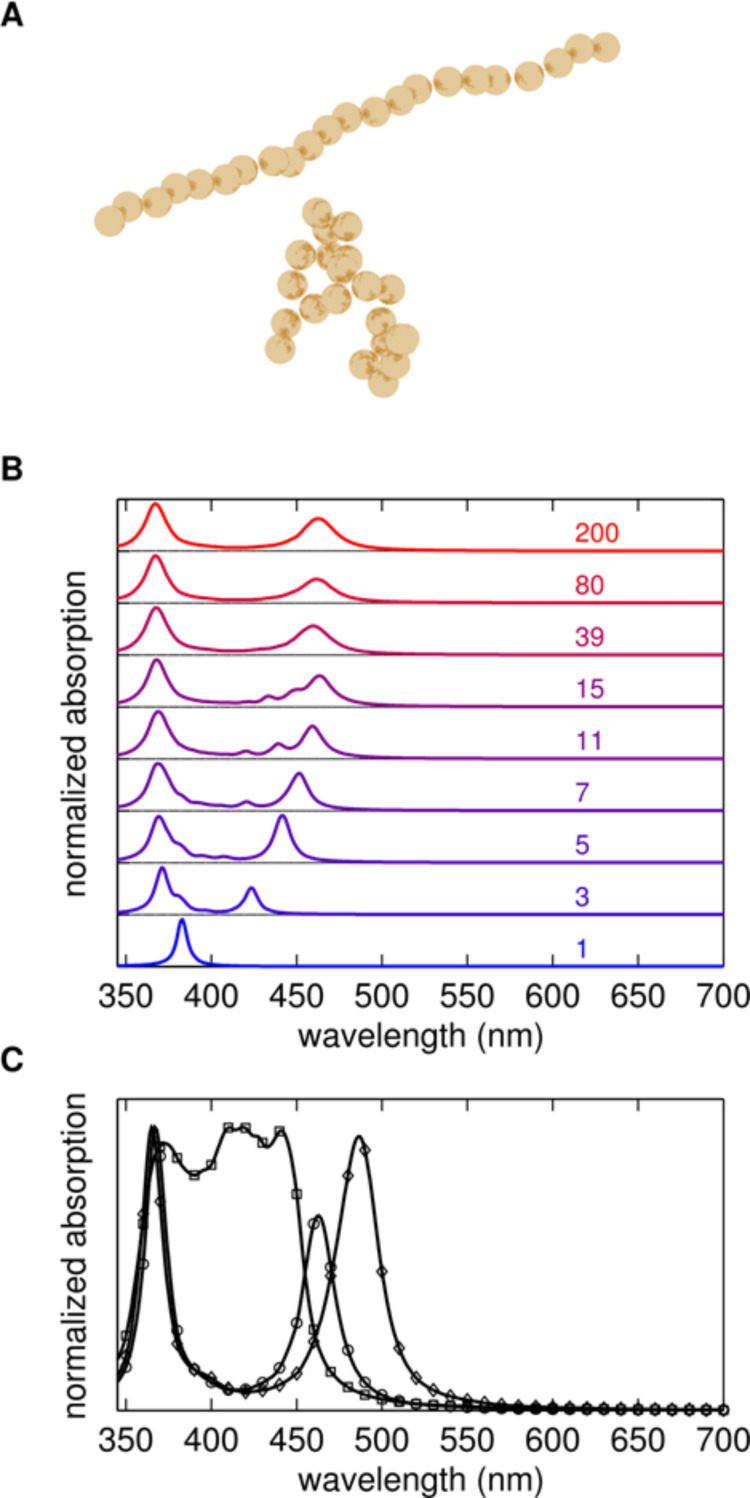
A) By controlling the allowed attachment point of new particles to the chain, anisotropic (top) and isotropic (bottom) aggregates are obtained. B) As the anisotropic aggregates grow longer, the transverse plasmon shifts to shorter and the longitudinal plasmon to longer wavelengths due to the interaction between the silver spheres. The number next to the curve gives the number of particles in the chains used to obtain the absorption spectrum. C) The spectra for isotropic (squares) and anisotropic (circles and diamonds) aggregates with 200 particles are distinctly different. For isotropic aggregates a broad band is observed without a clear longitudinal plasmon. For anisotropic chains the longitudinal plasmon shifts even further to the red when the inter-particle gap is reduced from 1.4 nm (circles) to 0.7 nm (diamonds) due to the stronger coupling.

With the addition of a ditopic ligand, this resonance broadens, as would be expected for the generation of nanoparticle aggregates. However, at some ligand ratios, a second resonance appears at longer wavelengths whose spectral position, intensity and broadness are influenced by the ligand-to-nanoparticle ratio. We attribute these spectral features to transverse and longitudinal plasmon resonances, respectively (see numerical simulations and Figure 2). The red shift in the longitudinal plasmon is due to plasmonic coupling between extremely closely spaced particles enhanced by the short length of the ligands (0.7–1 nm).

The appearance of a well-defined, distinct longitudinal band implies that the silver nanoparticles are forming anisotropic aggregates. For cysteine and DTT, at very high ligand-to-nanoparticle ratios, the transverse and longitudinal collapse into a single very broad resonance, which indicates a loss of structural anisotropy in the silver aggregates. The positions of the resonance bands are strongly dependent on the ligand-to-nanoparticle ratio (*r*), and are highly reproducible between experiments at the same ratio, as shown by the error bars in the inset of Figure 1, and in Figure S2 (Supporting Information File 1). In the absence of hydrogen-bonding ligands, uncoupled silver nanoparticles exhibit one band centred at 390 nm (Supporting Information File 1, Figure S1B), similar to the as-synthesized nanoparticles. Similarly, nanoparticles aggregated through the addition of salt to the solution demonstrate a small red shift, along with peak broadening as the salt concentration increases, but no second resonance appears, as expected for isotropic aggregation (Supporting Information File 1, Figure S3).

As Figure 1 shows, a threshold concentration of cysteamine, DTT, or cysteine is needed before a discrete longitudinal band can be observed. This threshold is the lowest for cysteamine and highest for cysteine. The particularly high threshold concentration for cysteine may be due to electrostatic repulsions given that cysteine is the only ligand with significant charge in these experiments. For all three ligands, broadening of the original nanoparticle resonance and an increase in scattering at higher wavelengths occur at lower ligand-to-nanoparticle ratios, indicating an increase in nanoparticle coupling prior to the formation of anisotropic assemblies that can support a longitudinal plasmon band.

The consistent and monotonous growth of *I*(λ_LP_)/*I*(λ_SP_) with increasing *r* for certain ratio intervals suggests that an estimate of ligand-coverage rates can be detected through plasmon coupling. Another significant feature of the spectra of these AgNPs assemblies is the retention of the transverse plasmon at UV wavelengths, with a slight blue-shift (2000 < *r* < 3250), while independently manipulating the intensity and position of the longitudinal band. This is also observed in strongly coupled assemblies, as shown in Figure 1A. This separation in the dependence of the two plasmonic modes on ligand interactions enables us to drive the coupled longitudinal mode to red-shifted wavelengths, extending the transmission of our assemblies throughout the visible region, which can be exploited for solar films and numerous energy saving applications. Luo et al. recently generated silver nanorods with high aspect ratio, which are transparent in the entire visible region using Pd decahedra as seeds [37].

In Luo’s work, the longitudinal band was pushed to the near infra-red region with nanorods of aspect ratios greater than 3.9. This is due to the energy dampening as the plasmon oscillation travels a longer distance on the rod surface [37]. We can accomplish a similar effect using a simple room-temperature process in aqueous solution, using ligand interactions to tune the optical properties of spherical nanoparticles through self-assembly. In our assemblies, the close proximity of AgNPs alongside with chain length modulation generated by varying the ligand concentration allows for a wider transparency in the visible window. This is most easily achieved with the cysteamine ligand, over a broad ratio range before the assemblies start to reinstate isotropy at elevated ligand concentrations. The regained isotropy is manifested by a broad significantly red-shifted band centered at 473 nm, as shown in Figure 1A.

Isotropy is restored at elevated ligand ratios in the cases of DTT and cysteine, where the spectra show a broad single plasmon red-shifted from 409 to 420 nm in AgNP-DTT and from 390 to 410 nm in the case of cysteine at high ligand-to-nanoparticle ratios. This suggests that when using DTT or cysteine, large aggregates lose their anisotropic character, and the two modes merge. This is consistent with our simulation results and previous observations where condensed isotropic aggregates were observed for AuNPs capped with mercaptoethanol (MEA) beyond an [MEA]/AuNPs ratio of 106:1 [38]. Those aggregates also showed a single broad band shifted 50 nm from that of the uncoupled particles. To the best of our knowledge, prior attempts to form anisotropic structures generated from silver nanoparticles have not shown distinct longitudinal and transverse plasmonic modes, in contrast to the observations reported here. For example, resorcinol-reduced silver nanoparticles were aggregated by heating, but the aggregates, despite their enhanced SERS signals, did not exhibit interesting spectral properties [39]. Likewise, AgNPs self-assembled by CTAB (cetyltrimethylammonium bromide) also did not reveal well-resolved anisotropy [25]. Our simulation results suggest that the inability of either of these AgNPs assemblies to support anisotropic optical modes could have been due to reduced polarizability, the short length of the nanoparticle chains involved in these structures, or insufficient inter-particle coupling, which greatly depends on the inter-particle gap.

**Figure 3:**
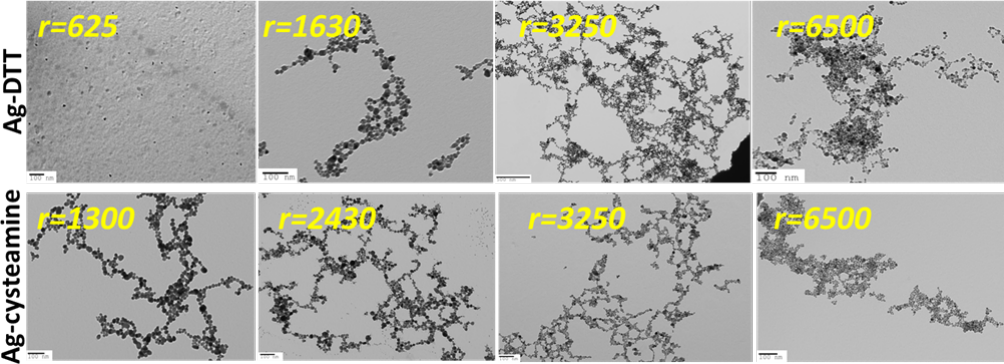
TEM micrographs of AgNPs modified by increasing ratios *r* of DTT and cysteamine ligands. Images show typical aggregates formed at the given ligand ratio.

The ligands with high affinity to silver chosen here are short and can potentially bind and drive particles together via H-bonding. It is worth noting that adding a certain amount of ligand at once does not yield the same spectral or morphological profiles as the gradual addition of the same amount of ligand. This observation suggests that assemblies form immediately in solution once the ligand is added. The assembly process happens spontaneously, and starts instantaneously before reaching a steady-state in less than 60 min. The spontaneity is likely enthalpy-driven due to dipole–dipole binding events that are exothermic. Although the assembly process is entropically not favourable, some entropy is regained through branching (Figure 3).

Figure 3 shows the development of aggregate size upon increasing the ligand concentration. At lower ligand-to-nanoparticle ratios, short-chained segments are predominant, and a number of branching points are observed, which are believed to mediate the plasmon flux. Some closed loops are also present. It is clearly evident that for AgNP–DTT, unlike AgNP–cysteamine, higher ligand ratios are needed to initiate assembly. This is consistent with the spectral evolution depicted in Figure 1. 20 µm long assemblies were noticed for *r* = 3250 (Supporting Information File 1, Figure S4). For all samples, TEM shows a fairly monodisperse nanoparticle size distribution, with an average diameter of 13 ± 3 nm, indicating that size and shape of the nanoparticles are not affected by the ligand-to-nanoparticle ratio.

One possible explanation for the linear chain-like structures observed at certain ratios of the ligands is the preferential binding of the ligands to certain facets of the silver nanocrystals. The differential binding affinity of ligands on different crystal faces makes both the nature of the ligand and the amount present critical in deciding the final crystal shape. Zhang et al. presented a mechanism of the 1D anisotropic growth of five-fold-twinned pentagonal silver crystals into silver wires when capped with polyvinylpyrrolidone (PVP) in the polyol synthesis. Their calculations showed that at high concentrations of PVP, the energy difference between the (100) and (111) facets is sufficiently reduced, eliciting a strain restriction, which in turn causes the 1D anisotropic growth into rods and wires [40].

**Figure 4:**
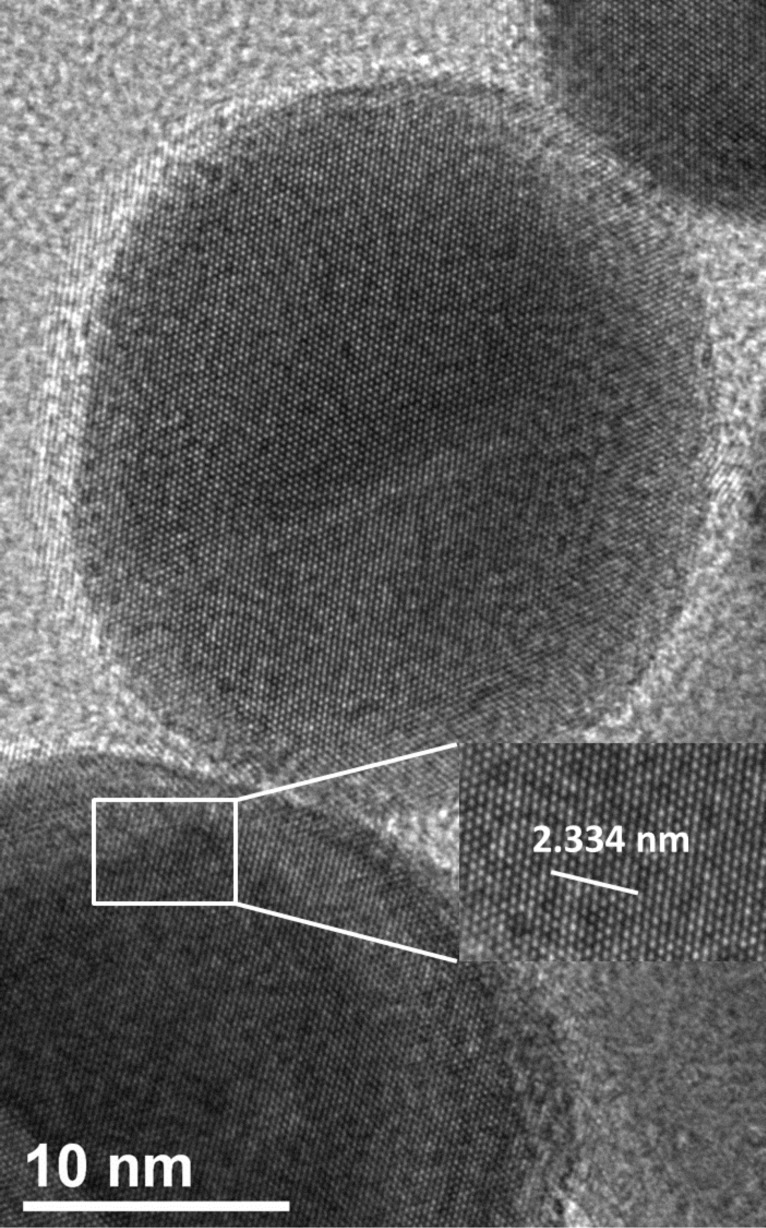
HRTEM image of AgNPs ensembles. The distance shown in the image corresponds to 10 × *d*. Here, *d* is the inter-planar distance, which was determined using the relation 
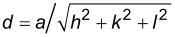
, where a is the lattice constant (4.08 Å for silver). It shows that the interacting facets are the [111] crystal facets, so the zone axis is 011.

A scenario inspired from this mechanism could explain the anisotropic growth of the AgNPs assembly in the presence of the ligands under study. At low ligand loading, the AgNPs are unsymmetrically capped due to the differential energy of their facets, and thus get linearly polarized, provoking dipolar interactions aligned and pre-set by ligand orientations. At high ligand ratios, the energy difference between the facets is lessened and the non-selective ligand loading evokes isotropic aggregate growth signed by a broad isotropic plasmon mode. Figure 4 presents a HRTEM image for two nanoparticles interacting through their [111] facets. The image reveals two major facets of nanoparticles under study, [111] and [200], with predominance of the former. A HRTEM image showing the [200] facet spacings is presented in Figure S5 (Supporting Information File 1).

**Figure 5:**
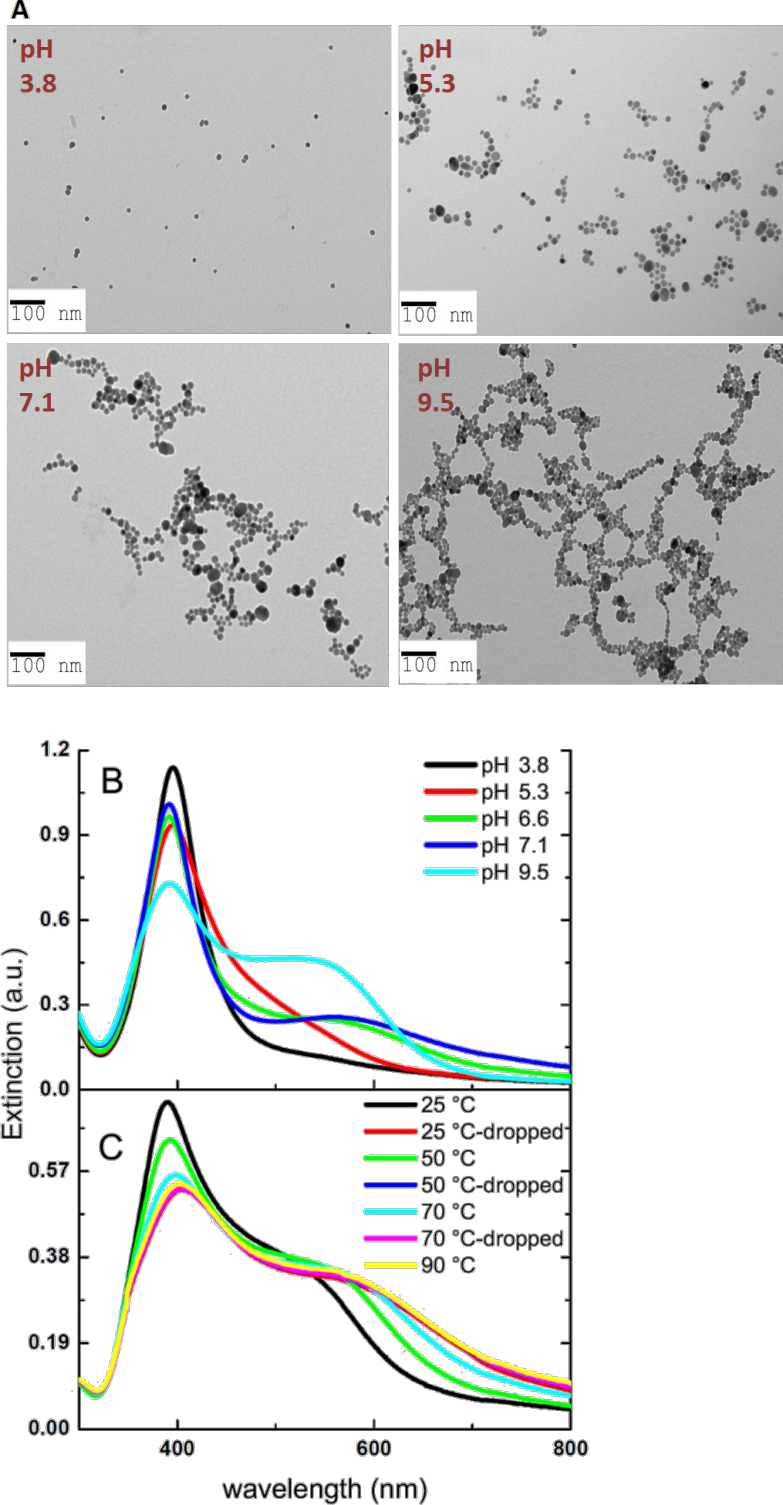
A) TEM images of AgNPs–cysteamine (*r* = 3250) under different pH conditions. B) Extinction spectra of AgNPs–cysteamine at 3 < pH < 10. C) Extinction spectra of AgNPs-cysteamine (r = 2000) at different temperatures.

Different pH values were used to test how H-bonding modulates the self-assembly and contributes to the stability of the assemblies. Protonating H-bonding moieties should reduce the ligand polarity and hence prevent nanoparticle assembly. Figure 5A clearly demonstrates this for AgNPs–cysteamine assembled at different pH values ranging between 3 and 10. Particle bundles begin to show up with an associated build-up of a longitudinal band at 550 nm as shown in Figure 5B. This pH-triggered assembly was found to be irreversible, probably due to the strong interaction between the ligands and the silver surface by a possible coordinate covalent bond. Kundu et al. have reported an influence of the pH value on the formation of silver nanochains reduced by 2,7-dihydroxynaphthalene upon microwave irradiation [26]. Increasing the temperature from 25 to 70 °C did not disrupt the AgNPs assembly as shown by the persistence of the anisotropic band despite a slight red-shift noticed as the temperature increased to 50 and then to 70 °C (Figure 5C).

Cooling the assemblies from 70 °C to room temperature did not restore the initial spectra, implying an irreversible change in assembly structure enforced at high temperatures. These robust AgNPs assemblies are comparably thermally resistant to previously described self-assembled gold nanoparticles [38] allowing for a broader blue-shifted bimodal optical tuning of plasmonic assemblies.

**Figure 6:**
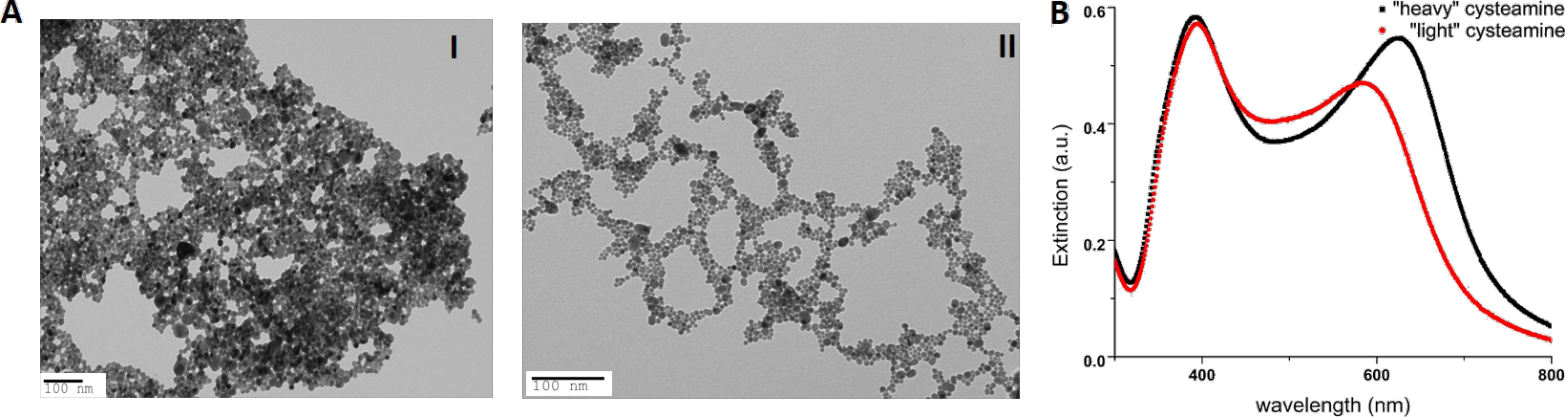
A) TEM micrographs of AgNPs modified with I: “heavy” cysteamine and II: “light” cysteamine (*r* = 3250). B) The corresponding extinction spectra of “heavy” cysteamine and “light” cysteamine-modified AgNPs.

To further examine the influence of hydrogen bonding on the assembly, the thiol and amine hydrogens of the cysteamine ligand were deuterated to obtain “heavy” cysteamine. The assembly and spectral response were characterized thereof and compared to those when “light” cysteamine was used instead. Heavy water was reported to be more structured than light water. It is also reported to be a weaker hydrogen bonding agent than light water due to quantum interference effects. The hydrogen bond in light water is 4% shorter than in heavy water [41]. Analogously, one expects that “light” cysteamine would be better solvated than “heavy” cysteamine. This in turn suggests that the inclusion of solvent molecules in the assembly process is possible. Figure 6A shows the distinct morphology of AgNPs aggregates in the presence of deuterated cysteamine. More intensely packed and dense aggregates are noticed, associated with greater coupling amongst the particles. The larger degree of coupling is reflected in the slight blue shift of the transverse mode and a more pronounced red-shifted longitudinal mode, which moves from 593 to 630 nm, as well as its higher intensity compared to the non-deuterated sample (Figure 6B). The two modes are better resolved upon increased coupling between the nanoparticles driven by the bigger size of the aggregates as well as the small inter-particle distance. The TEM images also show that AgNPs assembled by “heavy” cysteamine form aggregates which are more three-dimensional in nature, which means that branching starts to be omnidirectional.

A very clear example of solvent intervention in the mode of self-assembly of nanostructures was presented by Fava and co-workers. Side-to-side (via the CTAB-capped (100) facet) assembly of gold nanorods was favoured at a high content of the solvent tetrahydrofuran (THF), where THF is known to poorly solubilize CTAB. In contrast, end-to-end (via the polystyrene-capped (111) facet) assembly was noticed for more hydrophilic solvent combinations [42]. This helps to understand the role of water in the growth of the AgNPs–cysteamine assemblies under study. The poorer affinity of “heavy” cysteamine to the solvent (light water) resulted in more densely packed, three dimensionally assembled AgNPs, as indicated in Figure 6A-I. The kinetics of assembly in both cases was followed by monitoring the evolution of the absorption ratios Abs_700_/Abs_400_ over time. Similar kinetic profiles with no distinguishable features were observed (Supporting Information File 1, Figure S6).

To gain insight into the optical properties of the AgNPs aggregates, we carried out discrete-dipole approximation (DDA) simulations of random chains of silver spheres. Random chains of particles with different lengths were generated by attaching particles to the end of the growing aggregate with random growth direction as detailed in the Experimental section. The particle radius was chosen to be 7 nm, with an inter-particle distance of 0.7 and 1.4 nm, corresponding to the length of the ligand. Figure 2A shows examples of two realizations of the random chains. Linear aggregates are obtained by restricting the attachment point of a new particle to within a narrow solid angle defined by the growing chain. When the allowed solid angle is large, dense, isotropic aggregates are obtained.

Figure 2B shows the absorption spectra for chains with length ranging from 1 to 200 particles for linearly polarized incident light. The spectra were obtained by averaging over many realizations of the random structures. New realizations were added to the averaged spectrum until additional aggregates did not result in observable changes in the spectrum. In Figure 2B, anisotropic aggregates are considered (see above and Experimental section). We observe that the dipolar plasmon resonance of an isolated silver particle in water at approximately 380 nm wavelength is split into transverse (short wavelength) and longitudinal (long wavelength) resonances. The transverse plasmon blue-shifts slightly as the length of the chains is increased in agreement with what is expected for mode-splitting due to the coupling between the particles. The longitudinal plasmon shifts significantly to longer wavelengths, with the shift starting to saturate at 100 particle chain lengths.

The distinct long-wavelength peak observed in the experiments is attributed to the longitudinal plasmon in the chains. Figure 2C shows the absorption spectrum for anisotropic aggregates with 1.4 and 0.7 nm inter-particle gap and for an isotropic aggregate with 1.4 nm gap. For the isotropic aggregate, the absorption spectrum is broadened but no distinct bands are observed. For the isotropic aggregates the spectra show a clear longitudinal band, which shifts to longer wavelengths as the nanoparticles are brought closer and the inter-particle coupling is increased. The experimental spectra for AgNPs capped with the different ligands showed a red shift consistent with very small inter-particle spacing. The close spacing suggested by the observed red shifts agrees with the TEM micrographs, which show distances of less than a nanometre between neighbouring particles.

**Figure 7:**
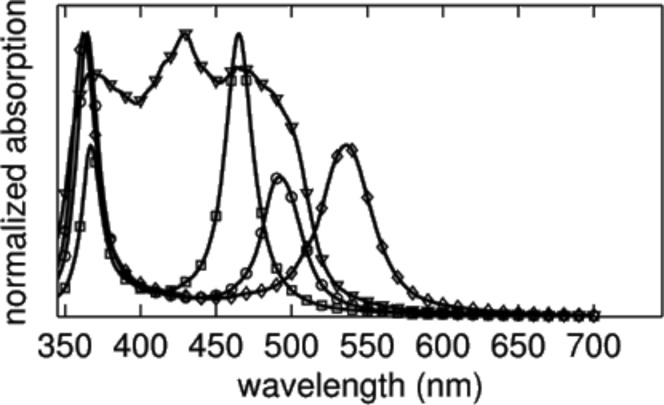
If the quasi-static polarizability is scaled by a factor of 1 (squares), 1.2 (circles) and 1.4 (diamonds) a significant red shift of the longitudinal plasmon for anisotropic aggregates follows due to the increased inter-particle coupling. For isotropic aggregates (triangles) the spectra remain broad. Aggregates with 200 particles were considered here.

The ligand layer is expected to result in a change of the polarizability due to the modified dielectric environment of the particles. In order to study the influence of this, we calculated the absorption spectra for aggregates of particles with a polarizability scaled from the value given by the quasi-static expression (see Experimental section). Figure 7 shows the influence of an increase of the particle polarizability by 20 or 40%. For increasing polarizability, the longitudinal plasmon for anisotropic aggregates shifts further to longer wavelengths. For isotropic aggregates, the broadening of the spectrum extends to longer wavelengths but no distinct resonances appear.

The calculated spectra clearly show how only anisotropically assembled aggregates result in the long-wavelength peak observed in experiments. For isotropic aggregates, the spectrum broadens but no distinct resonances are observed. The position of the longitudinal plasmon resonance depends sensitively on the inter-particle distance and the dielectric environment around the particles. Our simulation results are in good agreement with the experimental data and support the interpretation that the particles assemble in linear chains.

## Conclusion

A facile and quick self-assembly of AgNPs into highly anisotropic structures is presented using short ditopic ligands that are less than 1 nm in length. The degree of self-assembly can be readily dictated by the ligand concentration, pH and solvent, thus tuning the resulting optical properties. The assembled silver structures produce highly anisotropic UV–visible spectra reminiscent of high aspect ratio 1D nanoparticles such as rods. While such structures have been observed for gold nanoparticles, the greater reactivity of silver has made its controlled assembly more difficult. This degree of anisotropy reported here, with clearly separated resonances has not been previously demonstrated with assembled structures based on silver nanoparticles. For a certain window of ligand-to-nanoparticle ratio, the nanoparticles preferentially assemble through their [111] facets into extended networks of chains, which result in spectral anisotropy, as revealed by the simulation data. A stronger spectral anisotropy is achieved by deuterating the ligand due to the formation of highly dense anisotropic silver aggregates, offering a feasible method of preparing competent SHG (second harmonic generation) and SERS-active substrates. Based on discrete-dipole approximation simulations the observed anisotropy results from coupled modes of the nanoparticles. Simulations point to the importance of chain length, inter-particle spacing, and polarizability in controlling the optical properties of nanoparticle assemblies. Experiments are currently underway to correlate experimental spectra of individual assemblies with simulation to gain further insight on how to tune optical properties in nanoparticle systems via self-assembly.

## Experimental

**Materials**: All chemicals were used without further purification. AgNO_3_ (>99%, Aldrich), NaBH_4_ (98%, Aldrich), (95%, Aldrich), DL-dithiothreitol (Biotechnology grade, Fischer), cysteamine (95%, Aldrich), L-cysteine (97%, Aldrich), and D_2_O (98%, Aldrich). Deionized water (ρ >18 MΩ·cm) was used and obtained using a Barnstead Diamond TII (Thermo Fisher) purification system and the pH value was adjusted using 100 mM HCl.

**AgNPs synthesis and assembly:** AgNO_3_ (10.0 μmol) was dissolved in 100 mL of water at room temperature and chemically reduced under stirring by slowly adding an excess of cold NaBH_4_ (0.012 g) giving the characteristic yellow color of AgNPs colloidal solutions. The colloid was left for at least 4 h to age before the assembling ligand was added. The pH of the solution ranged between 9 and 10 and remained in this range after adding the ligands. The assembly was triggered by adding one of the three ditopic ligand solutions (1 mg/mL): cysteamine, cysteine and dithiothreitol (DTT). The added volume varied between 10 µL and 500 µL to trigger different degrees of assembly. The pH value of the AgNPs solution was set before the addition of the ligand. The solutions were then left in the dark under ambient conditions. Assemblies using the deuterated cysteamine ligand were carried out similarly after leaving cysteamine in excess D_2_O overnight.

**UV–vis and TEM characterization**: UV–visible extinction spectra in the range of 200–800 nm region were collected using a Cary 100 Bio instrument inside UV-Cuvette micro cuvettes (Light path 10 mm). For the temperature-controlled measurements, temperature was set using a dual cell Peltier accessory (Cary instruments). Kinetic scans were also done and the growth of the longitudinal plasmon (LP) with time was analysed using MATLAB (Mathworks, Natick, Massachusetts, USA). The size, morphology and size distribution were characterized by TEM. The nanoparticle facets were determined after HRTEM imaging using the lattice constant of silver (0.408 nm). The as-prepared samples were freshly plated on 200 mesh carbon-coated copper grids (Canemco-Marivac, Lakefield, QC) with a carbon film thickness ranging between 30 and 50 nm for 5 min before wicking using filter paper to avoid evaporation-induced aggregations. Images were collected using a Tecnai 12 TEM at 120 kV. Analysis of NP size was carried out using ImageJ 1.43u (Wayne Rasband, National Institutes of Health, USA). The number of particles was estimated using the average size determined from TEM and the density of bulk silver (10.5 g/cm^3^).

**Discrete dipole approximation simulations**: Electrodynamic calculations were performed on random chains of silver spheres. Coordinates for chains of random silver nanoparticles were generated using MATLAB. Particles were added to the end of the chain starting from a single seed particle. The attachment point of a new particle was chosen randomly on the surface of the particle at the end of the chain. If the chosen point resulted in two particles intersecting each other a new random location was chosen. To study the influence of the aggregate shape on the extinction spectrum, the allowed attachment points of a new particle were restricted to within a solid angle around the direction defined by earlier particles in the chain. Both the size of the solid angle and the length of the chain segment defining the reference growth direction were used as parameters in simulations. The particle aggregates obtained in this way well resemble the structures in the experiments (Figure 2).

Each silver particle was represented by a single dipole with a polarizability given by the expression 4πε_2_r^3^[ε_1_(ω) − ε_2_]/[ε_1_(ω) + 2ε_2_], where ε_2_ is the permittivity of the medium surrounding the particle, ε_1_(ω) is the frequency-dependent permittivity of silver, and *r* is the particle radius. This expression for the polarizability follows from the quasi-static approximation and is accurate for particles much smaller than the wavelength [43]. For silver published values for the permittivity were used in the simulations [44]. All simulations were performed with water as the surrounding medium. For water a constant refractive index of 1.33 was used. Absorption spectra for the particle aggregates were calculated using an in-house developed program implementing the discrete-dipole approximation (DDA) method [45]. A similar approach as the method used here has been earlier successfully used to model DNA-assembled nanospheres [46].

## Supporting Information

Supporting Information features data about control experiments, kinetics of assembly, and additional TEMs.

File 1 Additional experimental data.
