# Supplementary material for: Tunable longitudinal modes in extended silver nanoparticle assemblies
Source: Beilstein J Nanotechnol. 2016 Aug 26;7:1219–28. doi: 10.3762/bjnano.7.113 (PMC5082350; doi:10.3762/bjnano.7.113)
Supplement: File 1 — Additional experimental data. [file Beilstein_J_Nanotechnol-07-1219-s001.pdf]

Supporting Information

for

Tunable longitudinal modes in extended silver nanoparticle assemblies

Serene S. Bayram,[1] Klas Lindfors[2] and Amy Szuchmacher Blum[*1]

Address: [1]Department of Chemistry, McGill University, 801 Sherbrooke Street West, Montreal, QC H3A 0B8, Canada and [2]Department of Chemistry, University of Cologne, Luxemburger Str. 116, 50939 Köln, Germany

Email: Amy Blum* - amy.blum@mcgill.ca

* Corresponding author

Additional experimental data

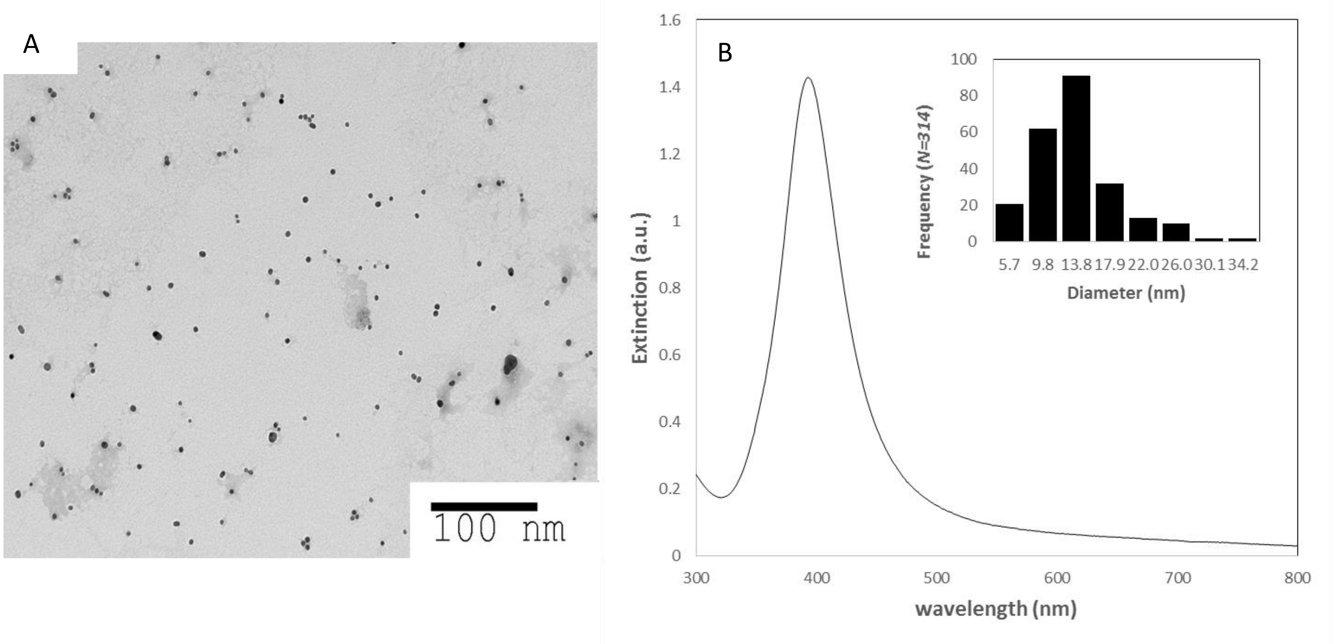

Figure S1: A) TEM image of as-synthesized AgNPs showing no sign of assembly. B) Extinction spectrum of the as-synthesized AgNPs showing the characteristic plasmon peak at 395 nm. Inset: Size distribution of AgNPs as measured from TEM showing an average diameter of 13.9 nm.

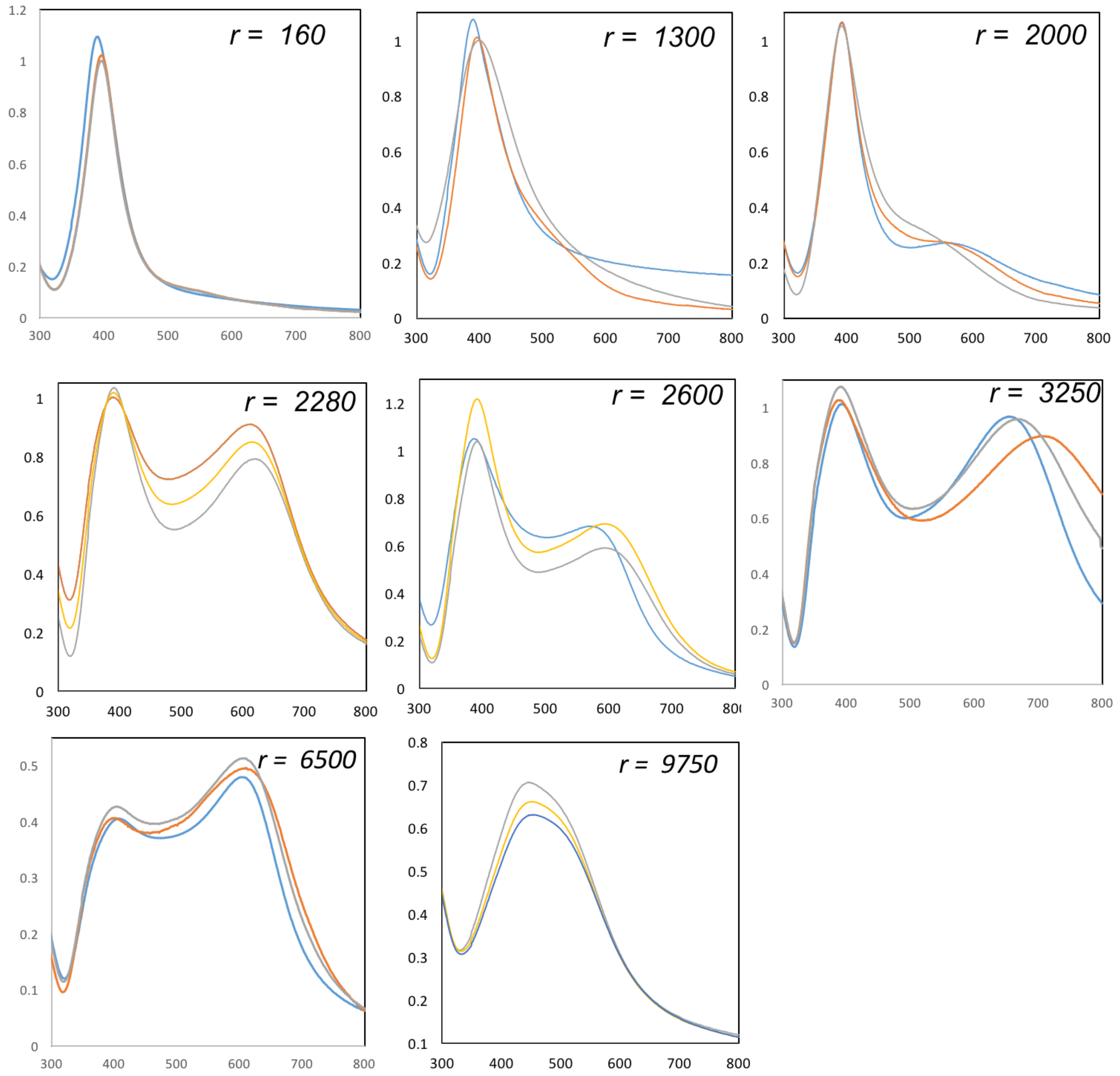

Figure S2: Extinction spectra replicates ($N = 3$) of AgNPs assembled under different cysteamine/AgNPs ratio conditions.

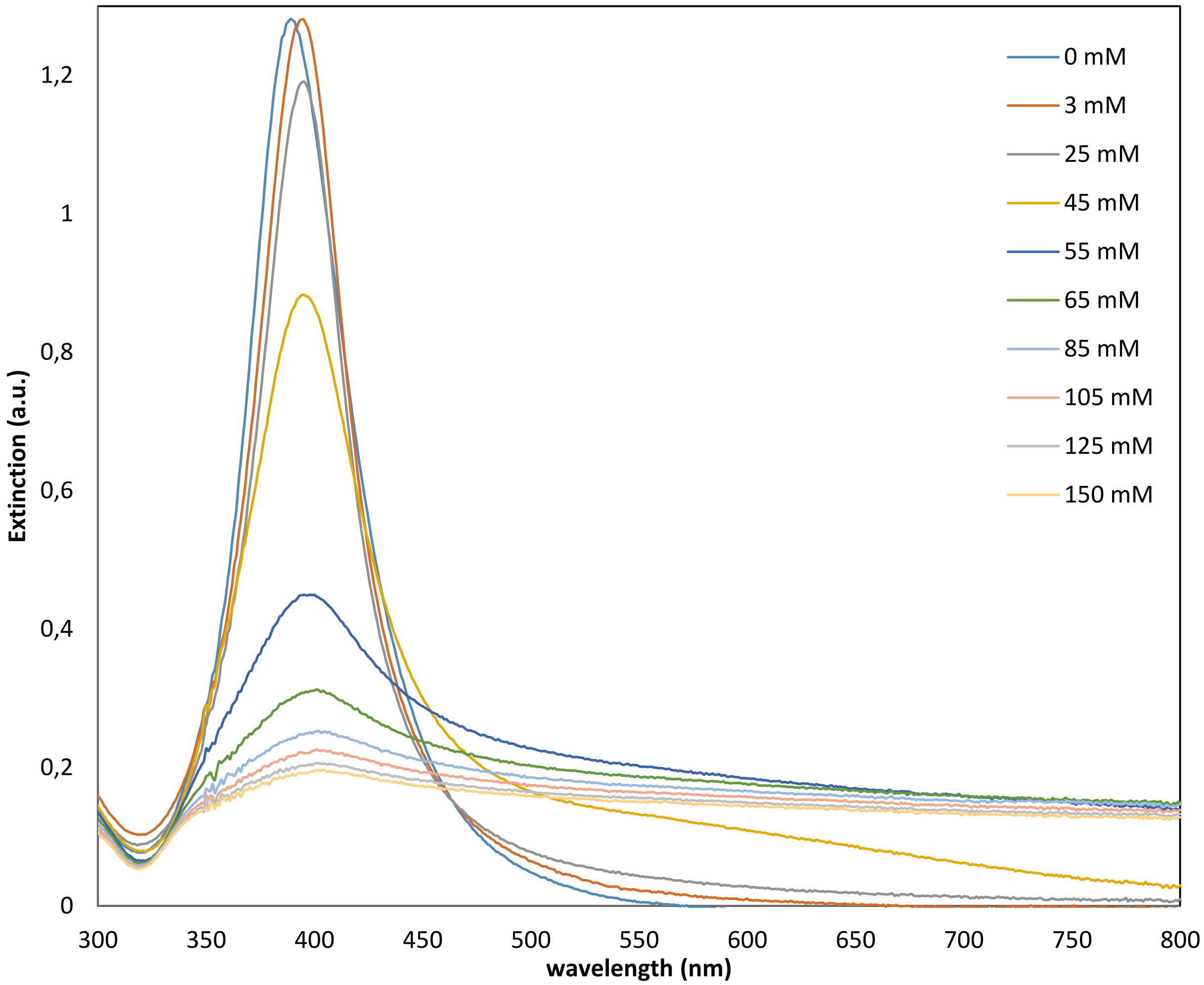

Figure S3: Extinction spectra of AgNPs aggregated by increasing concentrations of NaCl. At no concentration does a longitudinal mode appear. Increasing salt concentration causes a gradual red-shift from 398 to 406 nm with a collapse of the plasmon band as the nanoparticles start to crash out of solution.

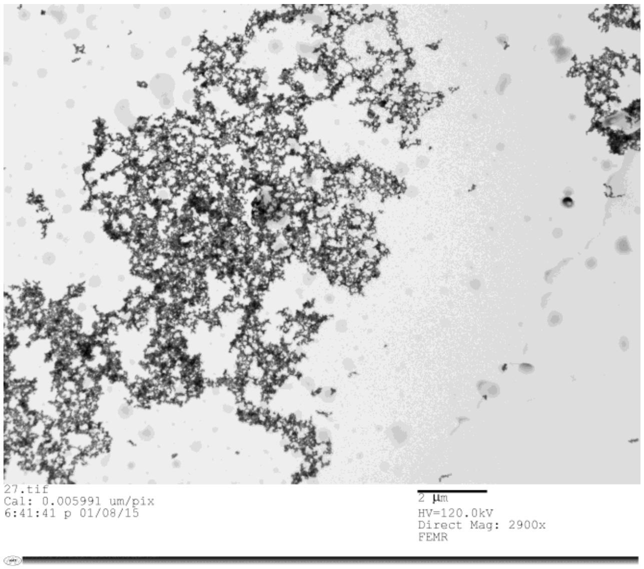

Figure S4: TEM showing the extended assembly of cysteamine-modified AgNPs (r = 3250). The micrograph shows that assemblies extend to more than 20 µm in length.

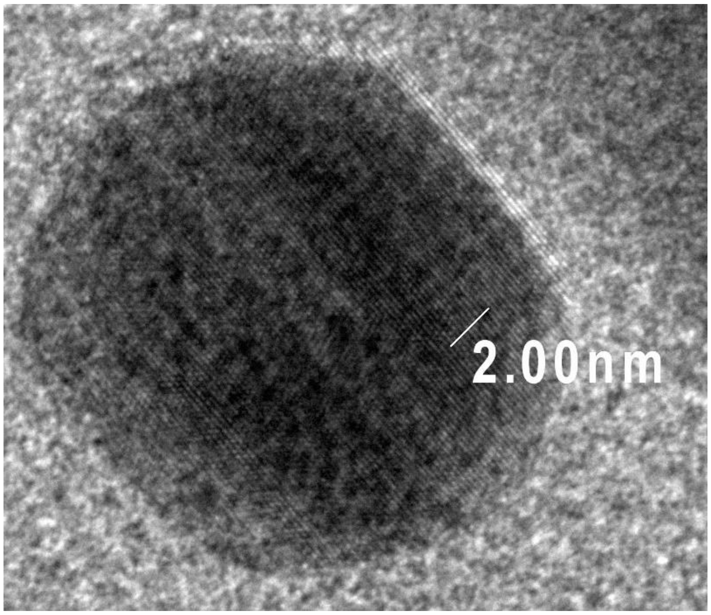

Figure S5: HRTEM of a silver nanoparticle showing the [200] facet. The distance shown in the image corresponds to $10 \times d$ (d = inter-planar distance) that was determined using the relation: $d = a/(h^2 + k^2 + l^2)^{1/2}$ where a is the lattice constant (4.08 Å for silver).

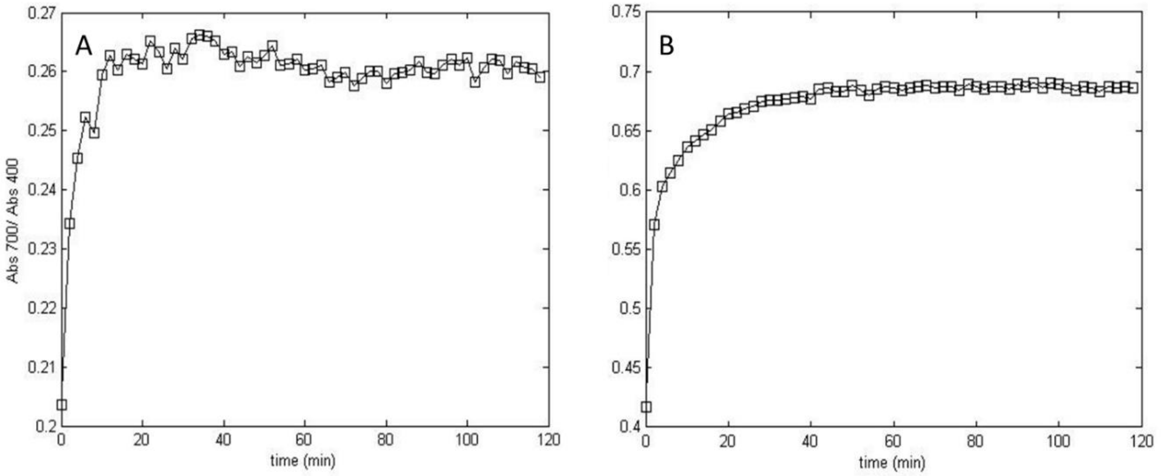

Figure S6: Kinetic profiles: Abs_{700}/Abs_{400} ratios for A) Ag–“light” cysteamine and B) Ag–“heavy” cysteamine.

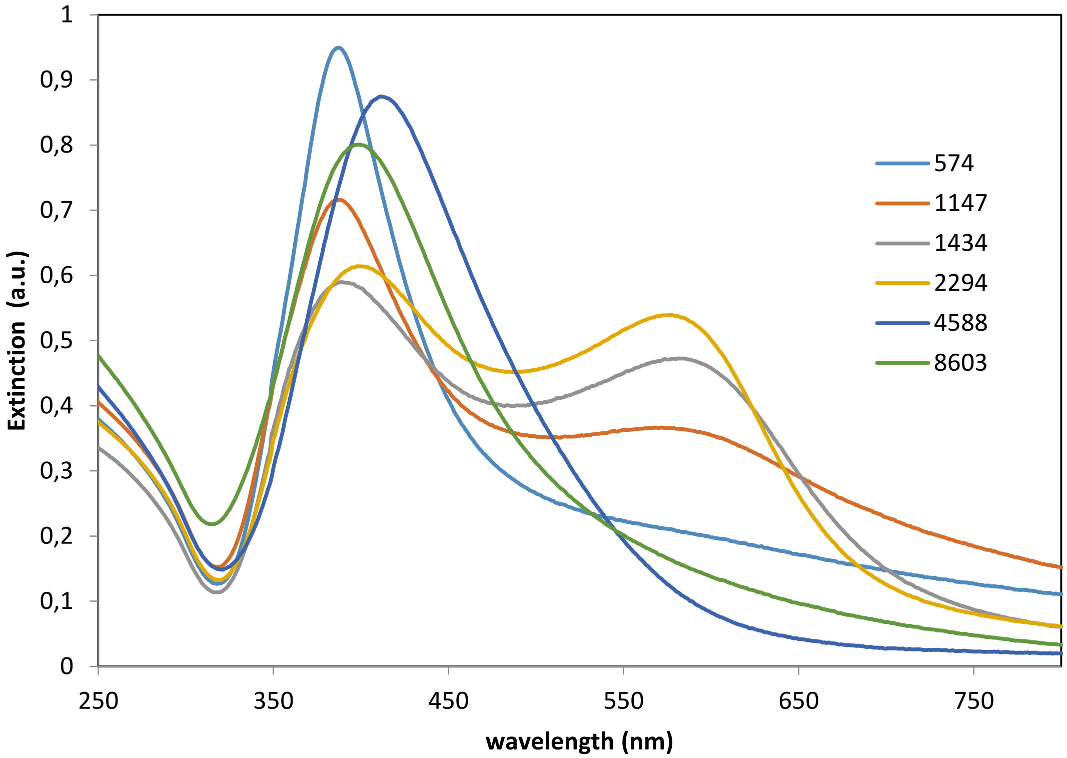

Figure S7: Extinction spectra of Ag–DTT with different *r*.

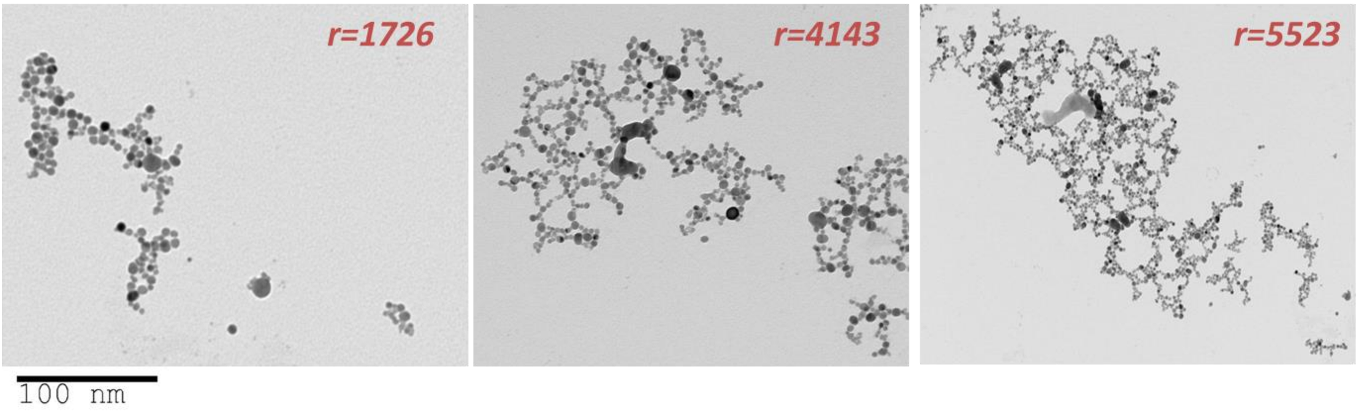

Figure S8: TEM images showing the growth of silver networks as the ratio of cysteine ligand to AgNPs increases.
